# Supplementary material for: Systematic Approaches to Study Eclipsed Targeting of Proteins Uncover a New Family of Mitochondrial Proteins
Source: Cells. 2023 Jun 5;12(11):1550. doi: 10.3390/cells12111550 (PMC10252432; doi:10.3390/cells12111550)

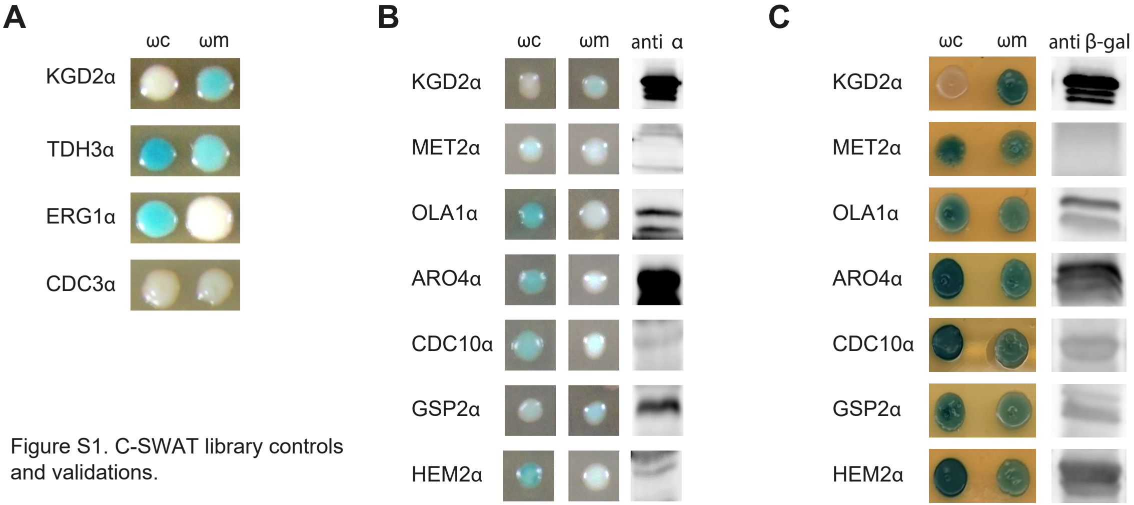

Figure S1. C-SWAT library controls and validations.

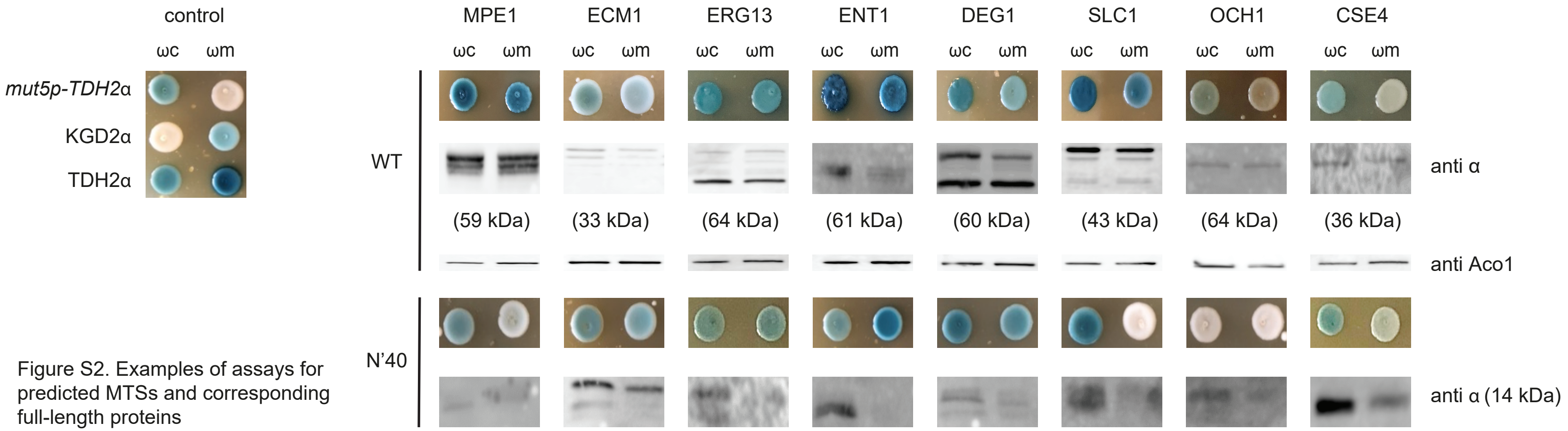

Figure S2. Examples of assays for predicted MTSs and corresponding full-length proteins

Figure S3. Tdh2/3 protein expression. Yeast strains harboring the indicated plasmids were grown on galactose media.

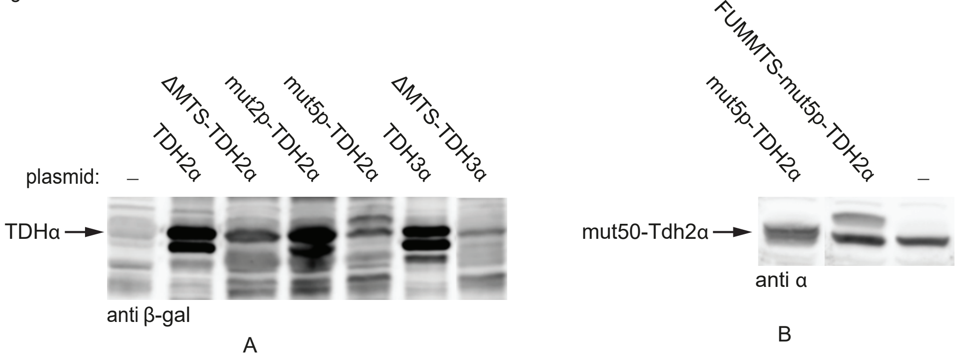

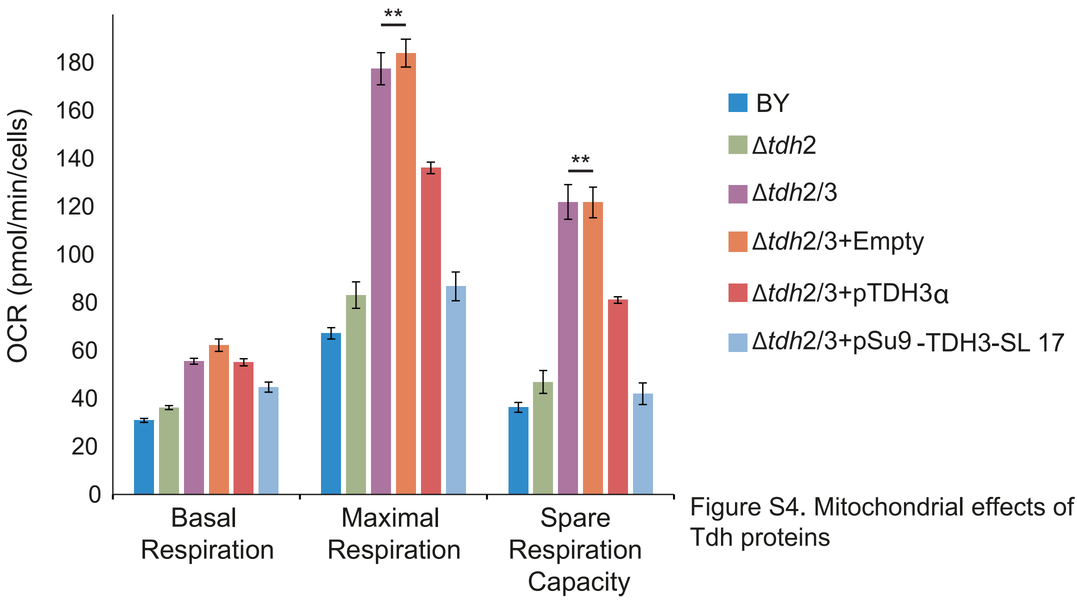

Figure S4. Mitochondrial effects of Tdh proteins

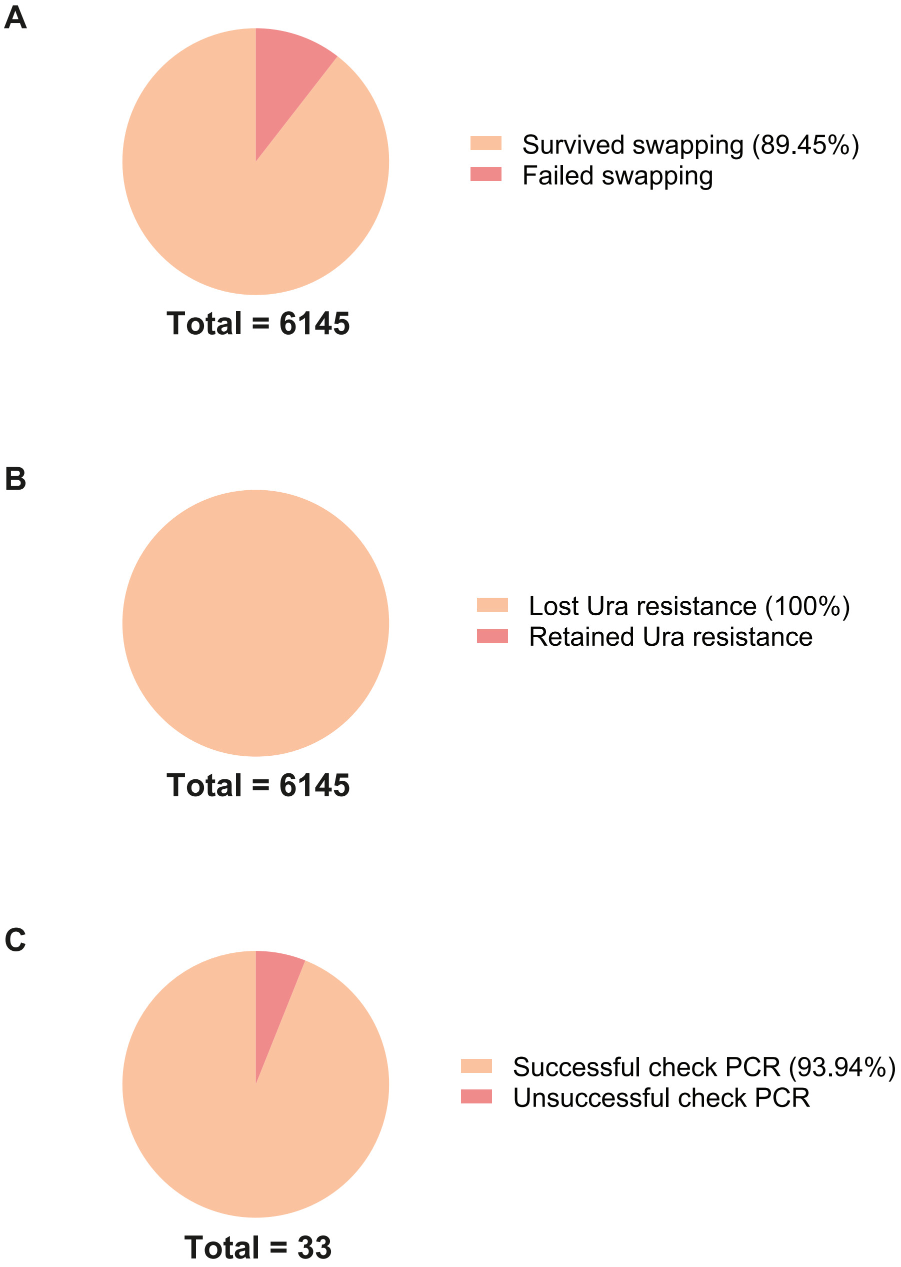

Figure S5. Evaluation of C-tagged alpha library SWATting efficiency and integrity.

Table S1. C-SWAT alpha tagged library results summary

*N**	Parameter	Reference
386	**Total blue colonies with ωm	This study
31 (of 386)	Over-expressed C-SWAT proteins for validation	This study
176/386	Previously alleged mitochondrial proteins	Previous studies/screens
116/386	Membrane proteins	GO annotation database
73/116	Membrane proteins not reported as mitochondrial	GO annotation database
137/386	***Novel non-membrane mitochondrial matrix candidates	This study

*N - Represents the number of proteins in each group.

**Blue colonies with ωm is based on α-complementation with the mitochondrial ω fragment.

***386-176-73=137 Novel non-membrane mitochondrial matrix candidates

Table S2. C-SWAT screen

Database		This study - C-SWAT	
SGD Genome database		α-complementation over expressed proteins (validation screen)	
ORF	Name	ωc	ωm
YBL022C	PIM1		
YER141W	COX15		
YHR051W	COX6		
YOL001W	PHO80		
YBL099W	ATP1		
YGL040C	HEM2	B	B
YPL090C	RPS6A		
YMR108W	ILV2		
YLR355C	ILV5		
YHL021C	AIM17		
YMR189W	GCV2		
YOR176W	HEM15		
YER073W	ALD5		
YBR181C	RPS6B		
YDL191W	RPL35A		
YDR148C	KGD2	W	B
YIL070C	MAM33		
YDL136W	RPL35B		
YDR258C	HSP78		
YGL127C	SOH1	B	W
YOR232W	MGE1		
YKL029C	MAE1		
YDL208W	NHP2		
YML100W	TSL1		
YLR448W	RPL6B		
YBR221C	PDB1		
YIL125W	KGD1		
YAL044C	GCV3		
YMR083W	ADH3		
YHR037W	PUT2		
YGR167W	CLC1	B	B
YLR192C	HCR1		
YGL187C	COX4		
YOR374W	ALD4		
YFL037W	TUB2		
YOR136W	IDH2		
YLR048W	RPS0B		
YBL045C	COR1		
YOL135C	MED7	B	W
YIL094C	LYS12		
YLR304C	ACO1		
YER069W	ARG5,6		

YGR214W	RPS0A		
YEL017C-A	PMP2		
YIL051C	MMF1		
YBR127C	VMA2		
YBR249C	ARO4	B	B
YDR070C	FMP16		
YJL153C	INO1	B	B
YDR298C	ATP5		
YDR418W	RPL12B		
YML073C	RPL6A		
YJR009C	TDH2		
YGR192C	TDH3		
YGL134W	PCL10	B	W
YOR185C	GSP2	B	B
YOR187W	TUF1		
YBL064C	PRX1		
YIL145C	PAN6	B	B
YKR092C	SRP40		
YNL287W	SEC21		
YGL207W	SPT16		
YPR124W	CTR1		
YDL178W	DLD2		
YKL210W	UBA1		
YJL123C	MTC1		
YPL135W	ISU1		
YDL130W-A	STF1		
YBR084W	MIS1		
YDR497C	ITR1		
YPR033C	HTS1		
YPL106C	SSE1		
YOR065W	CYT1		
YNL288W	CAF40		
YKL085W	MDH1		
YDR127W	ARO1		
YNL284C	MRPL10		
YCL017C	NFS1		
YMR105C	PGM2		
YBR020W	GAL1	B	B
YPR160W	GPH1		
YNL071W	LAT1		
YOL061W	PRS5		
YGL068W	MNP1		
YKR059W	TIF1	B	B
YPR191W	QCR2		
YJL138C	TIF2		
YHR039C-A	VMA10		
YDR502C	SAM2		
YNL241C	ZWF1	B	B
YGR094W	VAS1		

YOR122C	PFY1		
YAL005C	SSA1		
YGL113W	SLD3		
YML124C	TUB3		
YLR089C	ALT1		
YLR150W	STM1	B	B
YIL133C	RPL16A		
YNR050C	LYS9		
YBR039W	ATP3		
YDL130W	RPP1B		
YOR375C	GDH1		
YIL053W	RHR2		
YOR332W	VMA4		
YBR019C	GAL10		
YNL061W	NOP2		
YER086W	ILV1		
YLR058C	SHM2		
YDL195W	SEC31		
YLR295C	ATP14		
YMR178W			
YOR293W	RPS10A		
YBR263W	SHM1		
YLL041C	SDH2		
YJR080C	FMP26/AIM24		
YPR036W-A			
YDR354W	TRP4		
YNL220W	ADE12	B	B
YLR180W	SAM1	B	B
YJR121W	ATP2		
YNR001C	CIT1		
YJL200C	ACO2		
YOR142W	LSC1		
YGR244C	LSC2		
YGL202W	ARO8		
YNL037C	IDH1		
YOL140W	ARG8		
YHR008C	SOD2		
YGR180C	RNR4		
YFL016C	MDJ1		
YOR198C	BFR1		
YJR016C	ILV3		
YMR116C	ASC1		
YGL031C	RPL24A		
YFL018C	LPD1		
YDL066W	IDP1		
YDR019C	GCV1		
YMR230W	RPS10B		
YBR126C	TPS1		
YJL060W	BNA3		

YER055C	HIS1		
YPL195W	APL5		
YMR072W	ABF2		
YPL262W	FUM1		
YCL009C	ILV6		
YGL137W	SEC27		
YPL059W	GRX5		
YER101C	AST2	B	W
YML120C	NDI1		
YLR369W	SSQ1		
YPR004C	AIM45		
YGL037C	PNC1		
YOL149W	DCP1		
YKL039W	PTM1		
YJR144W	MGM101		
YHR052W	CIC1		
YKL035W	UGP1		
YAL012W	CYS3		
YMR296C	LCB1		
YJL026W	RNR2		
YBR031W	RPL4A		
YDR116C	MRPL1		
YDR194C	MSS116		
YDR012W	RPL4B		
YBR018C	GAL7		
YDR382W	RPP2B		
YGL032C	AGA2	W	W
YNL096C	RPS7B		
YDR036C	EHD3		
YIL109C	SEC24		
YHL001W	RPL14B		
YJR153W	PGU1	B	W
YOR230W	WTM1		
YKL009W	MRT4		
YJL148W	RPA34		
YML051W	GAL80		
YDL022W	GPD1		
YBR252W	DUT1		
YOR354C	MSC6		
YOR039W	CKB2		
YOR221C	MCT1		
YPL188W	POS5		
YER146W	LSM5		
YGR086C	PIL1		
YPL075W	GCR1		
YER107C	GLE2		
YLR061W	RPL22A		
YGR204W	ADE3		
YOR020C	HSP10		

YDL181W	INH1		
YLR441C	RPS1A		
YLR044C	PDC1		
YBR251W	MRPS5		
YJL133C-A			
YBL015W	ACH1		
YNL185C	MRPL19		
YDR525W-A	SNA2		
YGL135W	RPL1B		
YGR286C	BIO2		
YDR447C	RPS17B		
YML024W	RPS17A		
YFR015C	GSY1		
YDR174W	HMO1		
YDL192W	ARF1		
YMR062C	ECM40		
YGL173C	XRN1		
YDR032C	PST2		
YGR027C	RPS25A		
YBL089W	AVT5	B	W
YML063W	RPS1B		
YLR333C	RPS25B		
YDL131W	LYS21		
YER131W	RPS26B		
YMR226C	TMA29		
YLR258W	GSY2		
YFR032C-A	RPL29		
YCR053W	THR4		
YPL271W	ATP15		
YBR084C-A	RPL19A		
YNL121C	TOM70		
YBR121C	GRS1		
YBL027W	RPL19B		
YKL016C	ATP7		
YDR098C	GRX3		
YNL189W	SRP1		
YDL133C-A	RPL41B		
YNL243W	SLA2		
YGR207C	CIR1		
YDL184C	RPL41A		
YHR089C	GAR1		
YER056C-A	RPL34A		
YLR133W	CKI1		
YOR098C	NUP1		
YAR002W	NUP60		
YGR082W	TOM20		
YNL251C	NRD1		
YBL072C	RPS8A		
YNL031C	HHT2		

YGR124W	ASN2		
YMR300C	ADE4	B	B
YER048W-A	ISD11		
YML028W	TSA1		
YNL255C	GIS2		
YLL026W	HSP104		
YDR155C	CPR1		
YER024W	YAT2		
YHR017W	YSC83		
YJL020C	BBC1		
YCR088W	ABP1		
YNL084C	END3		
YLR114C	AVL9		
YCL040W	GLK1		
YPL004C	LSP1		
YJR076C	CDC11		
YBL047C	EDE1		
YDR529C	QCR7		
YGR254W	ENO1		
YDR129C	SAC6		
YJR007W	SUI2		
YGL062W	PYC1		
YML086C	ALO1		
YOL039W	RPP2A		
YBR218C	PYC2		
YFR053C	HXK1		
YHR174W	ENO2		
YIL093C	RSM25		
YBR025C	OLA1	B	B
YGL238W	CSE1		
YPR002W	PDH1		
YDL182W	LYS20		
YIL041W	GVP36		
YPR035W	GLN1		
YLR347C	KAP95		
YLL001W	DNM1		
YCR002C	CDC10	B	B
YDR033W	MRH1		
YLR337C	VRP1		
YHR107C	CDC12		
YNL015W	PBI2		
YGR218W	CRM1		
YPL118W	MRP51		
YDR428C	BNA7		
YOR089C	VPS21		
YER091C	MET6		
YOR285W	RDL1		
YGR178C	PBP1		
YDR050C	TPI1		

YPL037C	EGD1		
YBR011C	IPP1		
YJR145C	RPS4A		
YDL055C	PSA1		
YBL032W	HEK2		
YDL160C	DHH1		
YBL076C	ILS1		
YOR061W	CKA2		
YDL171C	GLT1		
YNR016C	ACC1		
YNL104C	LEU4		
YMR080C	NAM7		
YNL277W	MET2	B	B
YDR517W	GRH1		
YBL054W	TOD6		
YIL078W	THS1		
YHR135C	YCK1		
YNL016W	PUB1		
YDR381W	YRA1		
YER036C	ARB1		
YOR298C-A	MBF1		
YBR072W	HSP26		
YOR042W	CUE5		
YPL028W	ERG10		
YER025W	GCD11	B	B
YPR163C	TIF3		
YMR229C	RRP5		
YMR205C	PFK2		
YKL094W	YJU3		
YDR432W	NPL3		
YBL003C	HTA2		
YDR225W	HTA1		
YOR027W	STI1	B	B
YIL033C	BCY1		
YOR204W	DED1		
YNL134C			
YGR240C	PFK1		
YPL231W	FAS2		
YDR341C			
YJR109C	CPA2		
YOL123W	HRP1		
YNL004W	HRB1		
YLR399C	BDF1		
YGR159C	NSR1		
YML008C	ERG6	B	B
YKL152C	GPM1		
YNL138W	SRV2		
YKL067W	YNK1		
YPL061W	ALD6		

YJL057C	IKS1	W	W
YER177W	BMH1		
YDR099W	BMH2		
YJL080C	SCP160		
YJL201W	ECM25		
YLR372W	ELO3		
YMR183C	SSO2	W	W
YGR264C	MES1		
YDR353W	TRR1		
YGL105W	ARC1		
YIL062C	ARC15		
YGL245W	GUS1		
YLR370C	ARC18		
YDR023W	SES1		
YGL066W	SGF73	B	W
YPL169C	MEX67		
YDR171W	HSP42		
YPR041W	TIF5		
YCL037C	SRO9		
YML074C	FPR3	B	B
YDR172W	SUP35		
YLR175W	CBF5		
YIR006C	PAN1		
YOR310C	NOP58		
YLR060W	FRS1		
YHR183W	GND1		
YLL024C	SSA2		
YLR197W	NOP56		
YCR012W	PGK1		
YGL256W	ADH4		
YBL039C	URA7		
YLR449W	FPR4		
YDL215C	GDH2		
YPL190C	NAB3		
YMR012W	CLU1		
YER052C	HOM3		
YGR155W	CYS4		
YIL074C	SER33		
YMR243C	ZRC1		
YDL029W	ARP2		
YOL086C	ADH1		
YFR024C-A	LSB3		
YAL035W	FUN12		
YDR035W	ARO3		
YJR104C	SOD1		
YLR249W	YEF3		
YDL229W	SSB1		
YNL209W	SSB2		
YCL027W	FUS1		

YER151C	UBP3		
YDL185W	VMA1		
YOR317W	FAA1		
YGL049C	TIF4632		
YLR335W	NUP2		
YMR309C	NIP1		
YCR030C	SYP1		
YBR230C	OM14		
YNL208W			
YMR186W	HSC82		
YPL240C	HSP82		

Database		ITRAQ	
SGD Genome database		α-complementation of ITRAQ candidates	
**ORF**	**Name**	**ωc**	**ωm**
YBR125C	PTC4	B	LB
YER144C	UBP5	B	B
YDR389W	SAC7	B	B
YJL057C	IKS1	W	W
YDL225W	SHS1	B	B
YJL061W	NUP82	B	W
YGR252W	GCN5	B	B
YGL066W	SGF73	B	W
YER101C	AST2	B	LB
YIR034C	LYS1	B	B
YLL029W	FRA1	B	B
YLR455W	PDP3	B	B
YNL240C	NAR1	B	B
YHR206W	SKN7	B	B
YGL058W	RAD6	B	B
YGL189C	RPS26A	B	B
YMR070W	MOT3	B	B

Table S3. ITRAQ 17 proteins examined in α-complementation as described in the text. "B" indicates blue colony with the indicated ω; "W" white colony with the indicated ω; and "LB" light blue colony with the indicated ω..

Table S4. MTS guided screen

Database		This study - MTS guided screen			
SGD Genome database		MTS (40aa) α-complementation screen		MTS guided - Full length protein α-complementation screen	
ORF	Name	ωc	ωm	ωc	ωm
YBR140C	IRA1				
YMR154C	RIM13				
YOL127W	RPL25	B	LB	B	W
YKL006C-A	SFT1				
YCL033C	MXR2				
YNL072W	RNH201				
YHR070W	TRM5				
YFL001W	DEG1	B	B	B	B
YLR389C	STE23	W	B	B	B
YKL101W	HSL1	B	W		
YPL221W	FLC1			B	B
YMR002W	MIX17	B	LB	B	LB
YMR251W-A	HOR7	B	W	B	W
YLR332W	MID2	B	LB	B	B
YPL048W	CAM1	B	B	B	B
YLR151C	PCD1	B	B	B	W
YCR092C	MSH3	B	LB	B	B
YIL008W	URM1				
YKL059C	MPE1	B	B	B	B
YJL126W	NIT2			B	B
YBR216C	YBP1				
YAL046C	BOL3/AIM1	B	B	B	B
YNL194C		W	W	B	B
YJL030W	MAD2			B	B
YGL173C	XRN1				
YNL219C	ALG9	B	W	B	W
YER131W	RPS26B	B	LB	B	B
YMR226C	TMA29				
YER087W	AIM10	W	B	W	B
YCR053W	THR4	B	B	B	B
YBR084C-A	RPL19A	B	LB	B	B
YBL027W	RPL19B	B	LB	B	B
YDR064W	RPS13	B	B	B	LB
YNL107W	YAF9	B	B	B	B
YHR089C	GAR1	B	W	B	B
YER056C-A	RPL34A	B	W	B	B
YLR133W	CKI1	B	W	B	LB
YAR002W	NUP60	B	W	B	B
YBL055C	Tat-D	B	B	B	B
YDR379C-A	SDH6			B	B
YDL161W	ENT1	B	B	B	B
YKL037W	AIM26	B	W	W	W

YER123W	YCK3	B	W	B	B
YDR388W	RVS167	B	B	B	B
YDL072C	YET3			B	B
YJL062W-A	RRG10				
YDR500C	RPL37B	B	LB	B	B
YBL072C	RPS8A	B	W	B	W
YNL031C	HHT2	B	LB	B	B
YKR035W-A	DID2			B	B
YER047C	SAP1			B	B
YDR233C	RTN1			B	B
YML130C	ERO1			B	LB
Q0130	OLI1			B	W
YDL183C				B	B
YLR113W	HOG1			B	B
YKL161C	KDX1	B	W	B	B
YDL204W	RTN2			B	B
YPL187W	MF_ALPHA_1				
YDR381C-A		B	B	B	B
YEL043W		B	B	B	B
YDR428C	BNA7			B	B
YBR273C	UBX7				
YHR080C					
YJR145C	RPS4A	B	LB	B	B
YFL033C	RIM15				
YDL020C	RPN4			B	W
YGL142C	GPI10				
YPL116W	HOS3			B	B
YDL153C	SAS10			B	B
YKR003W	OSH6			B	LB
YPR065W	ROX1			B	B
YPL230W	USV1			B	B
YNL076W	MKS1			B	B
YLL003W	SFI1			B	W
YOR336W	KRE5				
YOL031C	SIL1			B	W
YGR194C	XKS1			B	B
YJL037W	IRC18			B	W
YBL054W	TOD6			B	B
YPL127C	HHO1	B	LB	B	B
YOR278W	HEM4			B	B
YHR135C	YCK1	B	LB	B	B
YHR127W	HSN1				
YCR027C	RHB1	B	W	B	B
YOR229W	WTM2			B	B
YJR118C	ILM1			B	LB
YGL163C	RAD54			B	W
YMR081C	ISF1			B	W
YDR434W	GPI17				
YOL060C	MAM3			B	B

YIL010W	DOT5	B	B	B	B
YGL030W	RPL30			B	B
YDR492W	IZH1	B	W	B	W
YDR339C	FCF1	B	LB	B	B
YNL029C	KTR5			B	W
YBR119W	MUD1			B	W
YJL013C	MAD3			B	B
YML059C	NTE1				
YGR090W	UTP22				
YBR114W	RAD16	B	LB	B	LB
YMR175W	SIP18				
YLR243W	GPN3	B	W	B	B
YPR034W	ARP7			B	B
YNL331C	AAD14			B	B
YJR136C	TTI2			B	LB
YML049C	RSE1				
YDR287W	INM2			B	B
YPR144C	NOC4			B	B
YNL127W	FAR11	B	LB	B	LB
YLR214W	FRE1			B	W
YKL197C	PEX1			B	B
YNL154C	YCK2			B	B
YKL013C	ARC19			B	B
YDL063C	SYO1			B	B
YOL003C	PFA4			B	B
YNL132W	KRE33				
YMR069W	NAT4			B	LB
YDL052C	SLC1	B	W	B	B
YOR280C	FSH3			B	B
YMR266W	RSN1			B	W
YBR102C	EXO84	B	LB	B	B
YGR245C	SDA1			B	LB
YOR167C	RPS28A			B	W
YLR223C	IFH1				
YML026C	RPS18B			B	LB
YNL242W	ATG2				
YDL060W	TSR1	B	W	B	B
YML113W	DAT1			B	B
YMR101C	SRT1	B	B	B	B
YML038C	YMD8			B	LB
YER007C-A	TMA20	B	LB	B	B
YJR014W	TMA22			B	B
YNL118C	DCP2			B	B
YOR047C	STD1			B	W
YNL326C	PFA3	B	W	B	B
YPR103W	PRE2			B	LB
YLR429W	CRN1			B	B
YKL188C	PXA2			B	B
YBL003C	HTA2	B	W	B	B

YCL026C-A	FRM2			B	LB
YJL186W	MNN5	B	W	B	W
YDR126W	SWF1	B	B	B	W
YKR061W	KTR2			B	W
YML016C	PPZ1			B	W
YJL024C	APS3			B	B
YDR225W	HTA1	B	W	B	B
YCL063W	VAC17			B	B
YDR087C	RRP1	B	B	B	B
YKR037C	SPC34			B	LB
YIL159W	BNR1				
YDR459C	PFA5	B	W	B	B
YBR199W	KTR4	B	W	B	W
YBL067C	UBP13	B	B	B	B
YMR316W	DIA1	B	B	B	W
YJL068C	SFGH	B	B	B	B
YJL046W	AIM22	B	B	W	B
YNR061C		B	W	B	B
YLR360W	VPS38	B	B	B	W
YNL314W	DAL82	B	W	B	B
YPR040W	TIP41	B	B	B	B
YCL058W-A	ADF1	B	W	B	B
YBR059C	AKL1				
YIL085C	KTR7	W	W	B	W
YLR002C	NOC3			B	B
YDR014W	RAD61	B	LB	B	B
YBL092W	RPL32	B	W	B	LB
YML126C	ERG13	B	B	B	B
YJL031C	BET4	B	B	B	LB
YER128W	VFA1	B	W	B	LB
YLR264W	RPS28B	B	B	B	B
YGR031C-A	NAG1	B	B	B	W
YBR175W	SWD3	B	W	B	B
YOR094W	ARF3	B	B	B	B
YNL151C	RPC31	B	LB	B	B
YOR049C	RSB1	B	W	B	LB
YGR060W	ERG25			B	B
YIL002C	INP51	B	B	B	B
YOR252W	TMA16	B	B	B	B
YLR299W	ECM38	B	W	W	W
YDR302W	GPI11	B	LB	B	B
YLR443W	ECM7	B	W	B	W
YNL301C	RPL18B	B	W	B	B
YOL120C	RPL18A	B	W	B	LB
YLR116W	MSL5	B	B	B	B
YLR380W	CSR1			B	B
YDR421W	ARO80	B	W	B	B
YGL126W	SCS3	B	W	B	LB
YPL006W	NCR1	B	W	B	W

YMR143W	RPS16A	B	W	B	W
YDL083C	RPS16B	B	LB	B	B
YER006W	NUG1	B	LB	B	B
YOR092W	ECM3	B	W	B	W
YNL106C	INP52	B	B	B	B
YER157W	COG3	B	LB	B	B
YOR073W	SGO1	B	LB	B	LB
YJL006C	CTK2	B	B	B	B
YCL048W	SPS22	B	W	W	W
YJR055W	HIT1	B	LB	B	W
YDL214C	PRR2	B	LB	B	LB
YFR042W	KEG1	B	W	B	LB
YLR074C	BUD20	B	W	B	LB
YKL008C	LAC1	B	LB	B	B
YGL003C	CDH1	B	LB	B	LB
YFR012W	DCV1	B	W	B	W
YGR154C	GTO1	B	B	B	B
YHL003C	LAG1			B	B
YPR043W	RPL43A	B	W	B	W
YLR361C	DCR2	W	W	W	W
YHR056C	RSC30	B	W	B	W
YML111W	BUL2			B	LB
YOL040C	RPS15	B	W	B	W
YOL103W	ITR2	B	B	B	B
YIL069C	RPS24B	B	LB	B	W
YDL226C	GCS1				
YLR450W	HMG2	B	LB	B	B
YIL103W	DPH1	B	LB	B	W
YKL049C	CSE4	B	B	B	LB
YJR155W	AAD10	B	B	B	LB
YDL159W	STE7			B	B
YOL068C	HST1			B	B
YNL254C	RTC4	B	LB	B	B
YGR065C	VHT1	B	W	B	LB
YPR129W	SCD6			B	B
YLL045C	RPL8B	B	LB	B	B
YIL106W	MOB1	B	B	B	W
YDR333C	RQC1	B	LB	B	W
YKR076W	ECM4	B	B	B	B
YOR190W	SPR1	B	W	B	W
YER113C	TMN3	B	W	B	LB
YOL062C	APM4	W	B	B	B
YPL267W	ACM1	B	W	B	W
YPL147W	PXA1	W	B	B	B
YHL033C	RPL8A	B	W	B	B
YPR085C	ASA1	B	B		
YJL097W	PHS1	B	W	B	B
YKL180W	RPL17A	B	W	B	B
YDR329C	PEX3	B	W	B	B

YMR137C	PSO2	B	W	B	W
YDL098C	SNU23	B	LB	B	LB
YJL095W	BCK1	B	W		
YGL067W	NPY1	B	B	B	B
YML093W	UTP14	B	LB	B	B
YBL005W	PDR3	B	W	B	W
YER001W	MNN1	B	W	W	W
YLL010C	PSR1	B	B	B	B
YML031W	NDC1	B	B	B	B
YMR127C	SAS2	B	LB	B	W
YBL004W	UTP20	B	W		
YOR076C	SKI7			B	B
YKL160W	ELF1	B	LB	B	B
YJL183W	MNN11	B	LB	B	W
YDL088C	ASM4	B	LB	B	B
YPR199C	ARR1	B	W	B	W
YLR260W	LCB5			B	B
YHR156C	LIN1			B	B
YFR051C	RET2	B	B	B	B
YKL022C	CDC16	B	B	B	B
YAL059W	ECM1	B	B	B	LB
YMR168C	CEP3	B	LB	B	B
YCR091W	KIN82	B	LB	B	B
YNL186W	UBP10			B	B
YDR171W	HSP42			B	B
YBL063W	KIP1	B	W		
YML043C	RRN11	B	LB	B	W
YOL115W	PAP2	B	LB	B	B
YIL090W	ICE2	B	W	B	B
YFR027W	ECO1	B	W	B	LB
YPR175W	DPB2	B	B	B	B
YDR130C	FIN1	B	W	B	B
YPL079W	RPL21B	B	W	B	W
YFL034C-B	MOB2	B	LB	B	B
YPL092W	SSU1	B	LB	B	W
YPL249C-A	RPL36B	B	B	B	B
YHR014W	SPO13	B	W	B	LB
YNL162W	RPL42A	B	W	B	W
YOR137C	SIA1	B	W	B	W
YER093C	TSC11	B	W		
YKL171W	NNK1			B	B
YLR363W-A		B	LB	B	B
YBR156C	SLI15	B	W	B	B
YBR041W	FAT1	B	B	B	B
YDL139C	SCM3	B	W	B	B
YPL157W	TGS1	B	W	B	B
YIL052C	RPL34B	B	W	B	B
YNL224C	SQS1	B	W	B	B
YPR132W	RPS23B	B	W	B	W

YDR072C	IPT1	B	W	B	W
YGL038C	OCH1	B	W	B	W
YCL066W	HMLALPHA1	B	W	W	W
YCR040W	MATALPHA1	B	W	B	W
YIL074C	SER33			B	B
YGR103W	NOP7	B	W	B	LB
YBR165W	UBS1	B	B	B	W
YFR031C-A	RPL2A	B	W	B	W
YIL018W	RPL2B	B	W	B	W
YFR024C-A	LSB3			B	B
YGL058W	RAD6	B	W		
YGR202C	PCT1	B	LB	B	B
YIL014W	MNT3	B	W	W	W
YER002W	NOP16	B	W	B	B
YBR009C	HHF1	B	W	B	LB
YNL030W	HHF2	B	W	B	W
YCL027W	FUS1	B	W	B	B
YMR281W	GPI12	B	W	B	LB
YAL040C	CLN3	B	B	W	B
YKL078W	DHR2	B	W	B	LB
YOR255W	OSW1	B	B	B	B
YJL189W	RPL39	B	W	B	W
YLR185W	RPL37A	B	LB	B	LB
YBR010W	HHT1	B	W	B	LB
YKL068W	NUP100	B	B	B	B
YJR138W	IML1	B	LB	B	B

Table S5. α-complementation of predicted MTSs

N	Parameter/Result	MTS score	% of 201
313	Predicted MTS containing proteins (without membranal).	> 0.55	N/A
201	Successfully cloned predicted MTS containing proteins.	> 0.7	100
53/201	BB, Suspected dual.	> 0.7	26.3
50/201	BLB, Uncertain suspected dual.	> 0.7	24.8
4/201	WB, Suspected mitochondrial.	> 0.7	2.0
91/201	BW, Suspected not mitochondrial.	> 0.7	44.7
3/201	WW, Not detected by α-complementation	> 0.7	1.9

N - represents the number of candidate proteins in each group.

MTS score – MitoProtll (http://ihg.gsf.de/ihg/mitoprot.html).

BB - Blue with ωc and blue with ωm.

BLB - Blue with ωc and light-blue with ωm.

WB - White with ωc and blue with ωm.

BW - Blue with ωc and white with ωm.

WW - White with ωc and white with ωm.

Table S6. α-complementation of full-Length proteins (MTS guided screen).

N	Parameter/Result	MTS score	% of 277
313	Predicted MTS containing proteins (without membranal).	> 0.55	N/A
277	Successfully cloned predicted MTS containing proteins.	> 0.55	100
194/277	Screened for their predicted MTSs (Table 2).	> 0.7	70
163/277	BB, Suspected dual.	> 0.55	58.8
42/277	BLB, Uncertain suspected dual.	> 0.55	15.1
3/277	WB, Suspected mitochondrial.	> 0.55	~1
62/277	BW, Suspected not mitochondrial.	> 0.55	22.3
7/277	WW, Not detected by α-complementation.	> 0.55	2.5

N - represent the numbers of candidate proteins in each group.

MTS score – MitoProtll (http://ihg.gsf.de/ihg/mitoprot.html).

BB - Blue with ωc and blue with ωm.

BLB - Blue with ωc and light-blue with ωm.

WB - White with ωc and blue with ωm.

BW - Blue with ωc and white with ωm.

WW - White with ωc and white with ωm.

Table S7. Statistical analysis of conservation parameters

Basic residues

	N	median	mean	SD	SE
not mitochondrial	3440	1	1.298	1.907	0.03252
known mitochondrial	1050	2	2.61	2.488	0.0768
known dual	336	3	3.006	2.608	0.1423
MTS guided	166	4	4.145	3.248	0.2521
CSWAT (total)	385	1	2.434	2.776	0.1415
CSWAT(new soluble)	137	1	1.759	2.451	0.2094

Kolmogorov-Smirnov test

	not mitochondrial	mitochondrial	dual	MTS guided	CSWAT (total)
known mitochondrial	<0.0001				
known dual	<0.0001	0.0365			
MTS guided	<0.0001	<0.0001	0.0064		
CSWAT (total)	<0.0001	0.0522	0.0065	<0.0001	
CSWAT(new soluble)	0.4252	0.0004	<0.0001	<0.0001	0.0438

Mann Whitney test

	not mitochondrial	mitochondrial	dual	MTS guided	CSWAT (total)
known mitochondrial	<0.0001				
known dual	<0.0001	0.0168			
MTS guided	<0.0001	<0.0001	0.0004		
CSWAT (total)	<0.0001	0.0309	0.0004	<0.0001	
CSWAT(new soluble)	0.1065	<0.0001	<0.0001	<0.0001	0.0065

uHD

	N	median	mean	SD	SE
not mitochondrial	3776	4.351	4.562	2.29	0.03727
known mitochondrial	957	5.732	5.777	2.697	0.0872
known dual	337	5.927	6.109	2.869	0.1563
MTS guided	166	6.902	6.963	2.083	0.1617
CSWAT (total)	385	5.107	5.476	2.762	0.1408
CSWAT(new soluble)	137	4.579	4.895	2.545	0.2174

Kolmogorov-Smirnov test

	not mitochondrial	mitochondrial	dual	MTS guided	CSWAT (total)
known mitochondrial	<0.0001				
known dual	<0.0001	0.2918			
MTS guided	<0.0001	<0.0001	<0.0001		
CSWAT (total)	<0.0001	0.0384	0.0193	<0.0001	
CSWAT(new soluble)	0.2561	0.0016	0.0011	<0.0001	0.2081

Mann Whitney test

	not mitochondrial	mitochondrial	dual	MTS guided	CSWAT (total)
known mitochondrial	<0.0001				
known dual	<0.0001	0.1181			
MTS guided	<0.0001	<0.0001	0.0002		
CSWAT (total)	<0.0001	0.0389	0.003	<0.0001	
CSWAT(new soluble)	0.2253	0.0003	<0.0001	<0.0001	0.04

Net charge

	N	median	mean	SD	SE
not mitochondrial	3440	-3	-5.67	18.91	0.3224
known mitochondrial	1050	3	2.188	15.09	0.4655
known dual	336	1	0.9077	13.51	0.7369
MTS guided	166	3	2.94	15.58	1.209
CSWAT (total)	385	-3	-4.182	17.33	0.8832
CSWAT(new soluble)	137	-6	-4.796	19.1	1.632

Kolmogorov-Smirnov test

	not mitochondrial	mitochondrial	dual	MTS guided	CSWAT (total)
known mitochondrial	<0.0001				
known dual	<0.0001	0.0465			
MTS guided	<0.0001	0.2671	0.005		
CSWAT (total)	0.7568	<0.0001	<0.0001	<0.0001	
CSWAT(new soluble)	0.0729	<0.0001	<0.0001	0.0002	0.1998

Mann Whitney test

	not mitochondrial	mitochondrial	dual	MTS guided	CSWAT (total)
known mitochondrial	<0.0001				
known dual	<0.0001	0.0144			
MTS guided	<0.0001	0.8645	0.1271		
CSWAT (total)	0.2808	<0.0001	<0.0001	<0.0001	
CSWAT(new soluble)	0.7225	<0.0001	0.0002	<0.0001	0.3571

CAI

	N	median	mean	SD	SE
not mitochondrial	3437	0.231	0.2508	0.08301	0.001416
known mitochondrial	1048	0.236	0.2647	0.1019	0.003147
known dual	336	0.247	0.2894	0.1393	0.007601
MTS guided	166	0.232	0.2624	0.1099	0.008531
CSWAT (total)	385	0.312	0.3713	0.1695	0.008637
CSWAT(new soluble)	137	0.314	0.3788	0.1843	0.01575

Kolmogorov-Smirnov test

	not mitochondrial	mitochondrial	dual	MTS guided	CSWAT (total)
known mitochondrial	0.0001				
known dual	<0.0001	0.0199			
MTS guided	0.834	0.6426	0.0229		
CSWAT (total)	<0.0001	<0.0001	<0.0001	<0.0001	
CSWAT(new soluble)	<0.0001	<0.0001	<0.0001	<0.0001	0.8148

Mann Whitney test

	not mitochondrial	mitochondrial	dual	MTS guided	CSWAT (total)
known mitochondrial	0.0002				
known dual	<0.0001	0.0065			
MTS guided	0.7212	0.2497	0.0063		
CSWAT (total)	<0.0001	<0.0001	<0.0001	<0.0001	
CSWAT(new soluble)	<0.0001	<0.0001	<0.0001	<0.0001	0.8724

Dn/Ds

	N	median	mean	SD	SE
not mitochondrial	3250	0.2376	0.2749	0.1912	0.003355
known mitochondrial	985	0.1967	0.2317	0.1779	0.005668
known dual	324	0.1784	0.214	0.1646	0.009144
MTS guided	148	0.2379	0.2661	0.1734	0.01425
CSWAT (total)	342	0.1193	0.1565	0.1235	0.006681
CSWAT(new soluble)	112	0.1403	0.1759	0.1337	0.01264

Kolmogorov-Smirnov test

	not mitochondrial	mitochondrial	dual	MTS guided	CSWAT (total)
known mitochondrial	<0.0001				
known dual	<0.0001	0.2198			
MTS guided	0.9133	0.0139	0.0016		
CSWAT (total)	<0.0001	<0.0001	<0.0001	<0.0001	
CSWAT(new soluble)	<0.0001	0.0008	0.0762	<0.0001	0.3958

Mann Whitney test

	not mitochondrial	mitochondrial	dual	MTS guided	CSWAT (total)
known mitochondrial	<0.0001				
known dual	<0.0001	0.1001			
MTS guided	0.9257	0.0061	0.0005		
CSWAT (total)	<0.0001	<0.0001	<0.0001	<0.0001	
CSWAT(new soluble)	<0.0001	0.0006	0.0251	<0.0001	0.1956

Homologes

	N	median	mean	SD	SE
not mitochondrial	3440	26	20.87	11.17	0.1904
known mitochondrial	1050	27	22.24	10.9	0.3363
known dual	337	28	24.27	9.678	0.5272
MTS guided	166	28	23.39	10.21	0.7924
CSWAT (total)	386	30	27.26	7.207	0.3668
CSWAT(new soluble)	137	30	27.72	6.583	0.5624

Kolmogorov-Smirnov test

	not mitochondrial	mitochondrial	dual	MTS guided	CSWAT (total)
known mitochondrial	0.001				
known dual	<0.0001	0.033			
MTS guided	0.0007	0.1632	0.6721		
CSWAT (total)	<0.0001	<0.0001	0.0015	0.0015	
CSWAT(new soluble)	<0.0001	<0.0001	0.0008	0.0075	0.8679

Mann Whitney test

	not mitochondrial	mitochondrial	dual	MTS guided	CSWAT (total)
known mitochondrial	0.0009				
known dual	<0.0001	0.0041			
MTS guided	0.0086	0.2998	0.2996		
CSWAT (total)	<0.0001	<0.0001	<0.0001	<0.0001	
CSWAT(new soluble)	<0.0001	<0.0001	0.0007	0.0001	0.8965

PGL

	N	median	mean	SD	SE
not mitochondrial	3440	0.47	0.3688	0.1911	0.003259
known mitochondrial	957	0.29	0.3267	0.1898	0.006135
known dual	337	0.24	0.2911	0.1832	0.009979
MTS guided	166	0.285	0.3339	0.189	0.01467
CSWAT (total)	385	0.2	0.2497	0.1788	0.009115

Kolmogorov-Smirnov test

	not mitochondrial	mitochondrial	dual	MTS guided	CSWAT (total)
known mitochondrial	<0.0001				
known dual	<0.0001	0.0026			
MTS guided	0.0676	0.815	0.0041		
CSWAT (total)	<0.0001	<0.0001	0.018	0.0001	
CSWAT(new soluble)	<0.0001	0.0011	0.1426	0.0367	0.407

Mann Whitney test

	not mitochondrial	mitochondrial	dual	MTS guided	CSWAT (total)
known mitochondrial	<0.0001				
known dual	<0.0001	0.0037			
MTS guided	0.0202	0.8308	0.0306		
CSWAT (total)	<0.0001	<0.0001	0.0017	<0.0001	
CSWAT(new soluble)	<0.0001	0.0003	0.1078	0.002	0.6002

GFP intensity

	not mitochondrial	known mitochondrial	known dual	MTS guided	C-SWAT (total)	C-SWAT (new soluble)
N	3301	849	303	142	361	132
Median	17.58	17.76	18.47	18.43	46.9	35.37
Mean	33.17	35.41	98.67	39.01	149.8	136.3
Std. Deviation	84.97	84.14	365.2	60.33	367.8	342.2
Std. Error of Mean	1.479	2.888	20.98	5.063	19.36	29.79

Kolmogorov-Smirnov test

	not mitochondrial	mitochondrial	dual	MTS guided	CSWAT (total)
known mitochondrial	0.0187				
known dual	0.0002	0.1049			
MTS guided	0.1639	0.5718	0.2885		
CSWAT (total)	<0.0001	<0.0001	<0.0001	<0.0001	
CSWAT(new soluble)	<0.0001	<0.0001	<0.0001	<0.0001	0.451

Mann Whitney test

	not mitochondrial	mitochondrial	dual	MTS guided	CSWAT (total)
known mitochondrial	0.3072				
known dual	0.0034	0.0375			
MTS guided	0.402	0.6885	0.292		
CSWAT (total)	<0.0001	<0.0001	<0.0001	<0.0001	
CSWAT(new soluble)	<0.0001	<0.0001	<0.0001	<0.0001	0.3528

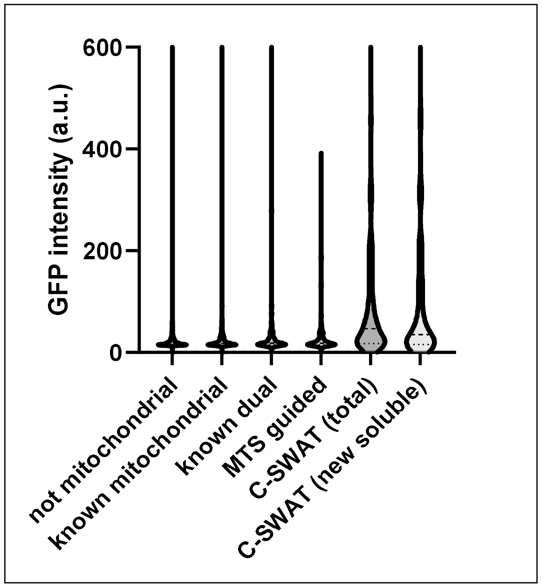

Supplement: Supplementary file 1 [file cells-12-01550-s001.zip › cells-2405362-supplementary.pdf]
